# A Patient-Centered Framework for Measuring the Economic Value of the Clinical Benefits of Digital Health Apps: Theoretical Modeling

**DOI:** 10.2196/18812

**Published:** 2020-10-30

**Authors:** Adam Powell, John Torous

**Affiliations:** 1 Payer+Provider Syndicate Boston, MA United States; 2 Division of Digital Psychiatry Beth Israel Deaconess Medical Center Harvard Medical School Boston, MA United States

**Keywords:** value, digital health, apps, payment models

## Abstract

**Background:**

As digital health tools such as smartphone apps evolve and enter clinical use, questions regarding their value must be addressed. Although there are scarce generalizable data on the value of health apps given their nascency and diverse use cases, it is possible to estimate the economic value of the clinical improvement they bring to patients using a quality-adjusted life-year (QALY)-based approach and generalized values from existing literature.

**Objective:**

This paper aims to provide a patient-centered framework for assessing the economic value of the clinical benefits delivered by digital health apps.

**Methods:**

We proposed a model based upon 5 levers: country-specific monetary value of a QALY, QALYs lost due to the condition, engagement rate of app users, average effect size of the app’s health impact, and duration of the app’s impact before remission.

**Results:**

Using 2 digital health apps from the United States and United Kingdom as examples, we explored how this model could generate country-specific estimates of the economic value of the clinical benefits of health apps.

**Conclusions:**

This new framework can help drive research priorities for digital health by elucidating the factors that influence the economic value.

## Introduction

As smartphone apps for health become more prevalent and their evidence base continues to expand, questions around the reimbursement and value of health apps are gaining importance. Today, insurers, health care organizations, and employers are signing contracts with app developers, even though the data and published literature on the economic value of health apps remain nascent. The existing data are either from small studies of single apps funded by the developers themselves, and thus introducing bias due to conflicts of interest, or from larger reviews [1]. Given the lack of economic evidence, there is a need for pragmatic models to guide informed decision making around pricing and determine the clinical value delivered by health apps.

The issue of measuring the value of digital health apps is of further importance, as digital health formularies are developed [2,3], and governments allocate taxpayer funds to cover costs associated with digital health tools [4,5]. Costs associated with apps are currently reimbursed using a variety of channels, including Current Procedural Terminology codes, device codes, and laboratory codes [6]. App users are also paying for the costs associated with apps directly, through a combination of one-time payments, in-app payments, subscription models, and participation in advertising [7]. However, some apps are not readily reimbursable within the existing frameworks, and thus app-specific reimbursement channels may need to be developed [8].

All economic activity, including the reimbursement of health apps, is about trade-offs; and higher-value interventions are typically preferred to lower-value interventions when resources are scarce. Generally, apps have a higher price point if they offer some form of human support, such as coaching, reflecting the additional costs associated with delivering that service. Given that human guidance within apps has been shown to be significantly associated with larger effect sizes, an analysis is needed to ensure that the cost of such guidance is outweighed by its benefits [9].

Value has been defined in the context of health care as outcomes relative to costs; when outcomes improve or costs decline, it suggests an improvement in value [10]. The items that are included in a value analysis depend upon the intended user of the analysis and thus will vary between patients, providers, health care systems, and payers. For example, if the intended user is a health care provider organization, which has based its decision to adopt a technology upon its own welfare, then the costs included in the analysis will only be those relevant to that organization. A framework for measuring the value delivered to a radiology department by a software, which helps the department detect anomalies more efficiently, listed the following elements: one-time direct costs, one-time costs of operational changes, ongoing change in direct costs, ongoing cost of operational changes, and ongoing change in downstream costs [11]. None of these costs are relevant in an analysis that takes a patient-centered perspective.

This paper aims to provide readers with a patient-centered framework for assessing the economic value of the clinical benefits delivered by digital health apps. Although patients also potentially receive value from nonclinical benefits, such as improved productivity at work, this paper strictly focuses on the valuation of the improvement in health outcomes. Value that accrues to other stakeholders, such as health care providers and payers, is outside the scope of this analysis. This approach has been chosen, as self-pay is the primary model of payment for many mental health apps. The approach is also appropriate for app evaluations made by a paternalistic payer whose primary objective is to maximize health benefits that patients achieve for a given level of spending (eg, a large government payer who does not consider increases in productivity or cost substitution benefits).

Although specific data on individual apps are often not available, there are now enough data from meta-analyses on the effect sizes of apps’ impacts on health and research on engagement to inform general models around value. Health care providers and payers are also impacted by the use of apps, but evaluating the financial impact caused by apps on these users is outside of the scope of this paper. These other stakeholders experience changes in one-time, ongoing, and downstream costs. The degree to which these changes are borne by health care providers or payers is determined by the nature of their contracts and the extent to which each is exposed to the cost of utilization.

## Methods

There are 2 main components in the outcome component of the value equation: (1) change in clinical outcomes and (2) change in financial outcomes. Although clinical outcomes are experienced as health, and not as money, they can be translated into financial terms. Many societies have in various ways indicated their willingness to pay for improvements in health as measured in quality-adjusted life-years (QALYs). A QALY is “a measure of the state of health of a person or group in which the benefits, in terms of length of life, are adjusted to reflect the quality of life,” where a year of perfect health is equal to 1 QALY [12]. In the United States, willingness to pay for a single QALY appears to be somewhere between US $50,000 to US $500,000, with a cutoff value of US $175,000 beyond which the Institute for Clinical and Economic Review no longer classifies an intervention as “low value” [13,14]. By mapping clinical outcomes to money, it is possible to measure values solely in monetary terms.

**Figure 1:**
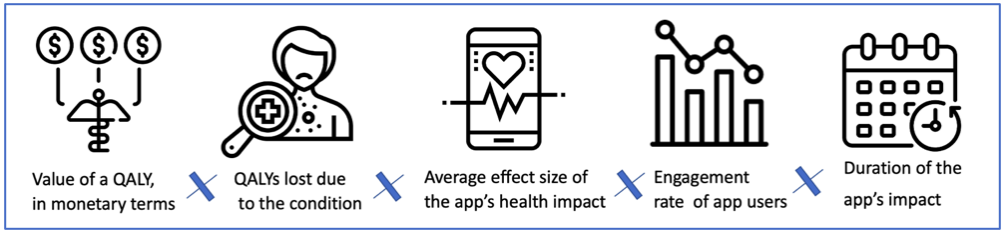
Methodology for estimating the economic value of the clinical benefits of digital health apps. Economic value of an app’s clinical benefits = country-specific monetary value of a QALY * QALYs lost due to the condition * engagement rate of app users * average effect size of the app’s health impact * duration of the app’s impact before remission.

As shown in Figure 1, the economic value of the clinical benefits delivered by an app is determined by the following 5 levers:

1. Country-specific monetary value of a QALY
2. QALYs lost due to the condition
3. Engagement rate of app users
4. Average effect size of the app’s health impact
5. Duration of the app’s impact before remission

## Results

As an example, we can estimate the value delivered by an app used in the United States for reducing depression. The data used below are derived from recent evidence, although assumptions must be made where the data are currently limited, not publicly available, or unclear. Estimates for the 5 levers of the model were derived as follows:

1. As previously mentioned, willingness to pay for QALYs in the United States appears to be somewhere between US $50,000 and US $500,000, per year, with US $175,000 per year serving as a potential cut-off for a low-value intervention [13,14].
2. The literature suggests that patients on average lose 0.159 QALYs per year from depression, based upon depression’s impact on EuroQOL 5 dimensions (EQ-5D) questionnaire scores [15]. Although a single number cannot capture the diversity of ways in which people are impacted by depression, this number offers an evidence-based estimate useful for modeling.
3. Health apps are often downloaded but are rarely used more than a few times. A recent study examined real-world data on app use to conclude that only 4% of users actually engage with apps meaningfully after 15 days [16], suggesting that few people receive an adequate “dose” of apps. The degree of engagement can be impacted through the use of human coaching or peer support or through app design [17,18].
4. Many app studies define a response as 50% reduction in symptoms. Studies on remission often also feature a 50% reduction in symptoms, which brings patients into a lower range of depression scores, indicating that patients may now experience lack of functional impairment related to the illness. Thus, as an estimate, it is reasonable to assume based on the current evidence that the effect size of apps for depression may offer up to a 50% reduction in symptoms [19].
5. There is little evidence on the long-term effects of mental health apps in sustaining benefits among users. Most studies feature no follow-up data, although some suggest mixed results, such as no impact at 3 months [20], while others suggest maintained benefit [19]. Assuming that these apps can yield a benefit at 3 months, we can use this number in our models.

When these 5 levers are considered together, we can form an estimate of the economic value of the clinical benefits delivered by an app for depression. Although the numbers used in the above 5 stages are estimates, they provide reasonable guidance and can be adjusted by the user for any particular app and health condition under consideration. Using these numbers, the following estimate of economic value can be generated:

US $175,000 per QALY × 0.159 QALYs lost per year of depression × 4% receiving effective dose × 50% reduction in symptoms × 0.25 years of improvement = US $139.13

The above example suggests that the economic value of the clinical benefit is US $139.13 per patient treated, US $11.59 per month if all users subscribe to the app for a year. Note that the outcomes delivered may achieve a higher valuation if nonclinical outcomes, such as enhanced wages at work due to greater productivity or savings within the health care system, are considered while developing an estimate. Nonetheless, on purely clinical grounds, the value delivered by an app addressing depression leads to a pricing that seems within the bounds of what is observed in the marketplace today.

Each of the numbers used in our example for a depression app will vary based upon the unique context at hand. For example, in a country with developing economy or a country more frugal with its health care resources is likely to place a lower value on a QALY than the one placed by the United States. For a second example, consider an app deployed in the United Kingdom, which has a user engagement rate that has been enhanced through the use of peer support. To further examine how these levers can change outcomes, the second example will explore an app for anxiety management, rather than depression support. Lever values are as follows:

1. In the United Kingdom, the government’s threshold for cost-effectiveness has been reported within the range of £20,000 to £30,000 per QALY (roughly US $25,000 to US $40,000 per QALY) [18].
2. The literature suggests that patients lose an average of 0.070 QALYs due to anxiety based on recent evidence from the EQ-5D questionnaire [15].
3. The engagement rate of app users can be increased to as high as 17% with the addition of peer support, and the rate will vary by app and health condition [16]. Although adding coaches or peers to encourage uptake can benefit the clinical outcomes of the value equation, these additions come with a trade-off of added ongoing costs.
4. The duration and durability of the health impacts of the app likely vary. For simplicity, we assume that the app has the same duration of impact as that of the previously examined depression support app (a 50% reduction in symptoms).
5. Similarly, we assume that the duration of the impact is the same as it was for the depression support app (3 months).

By altering 3 of the levers in the equation (reducing the value of a QALY to US $25,000, reducing the QALYs lost from the condition to 0.070, and increasing the engagement rate to 17%), we can estimate that the clinical value delivered by the anxiety management app with peer support in United Kingdom is as follows:

US $25,000 per QALY × 0.070 QALYs lost per year of anxiety × 17% receiving effective dose × 50% reduction in symptoms × 0.25 years of improvement = US $37.19

If we amortized the US $37.19 clinical benefit over a year, the value per month would be US $3.10.

## Discussion

As digital health apps mature, evidence-based pricing models have not kept pace with the market demands. Our model offers a simple, interpretable, and context-specific means to estimate cost and understand factors that may change the economic value of a digital health app. As the evidence for these apps continues to evolve, the results of this model will become more accurate. Given that many subscription-based depression support apps are currently priced at around US $12 a month, our model offers face validity.

The estimates provided by the model are imprecise and subject to some limitations. As illustrated by the examples, estimates will vary across countries due to national differences in parameter values (eg, the valuation of a QALY), even if the apps themselves remain unchanged. It is also possible that the parameters are not fully independent, and many do not have linear relationships. For instance, apps with higher effect sizes may have higher engagement rates, as people sense the effectiveness of those apps and remain more engaged. Furthermore, some apps may be outliers and have parameter values that deviate so substantially from other similar apps that the estimates of the proposed model are not representative. The effect size of a depression support app may differ between people or populations. These situations can be rectified if app-specific parameter estimates are used, rather than generalizations. As with all models, modelers must weigh the effort of obtaining more precise parameter values against the benefit of a more precise estimate.

When considering app evaluations in other contexts, it may be necessary to alter evaluation models in order to better address the context in which deployment is planned [21]. With the advent of personalized medicine in digital health, it may be possible to use digital biomarkers and other factors to identify the patients most likely to respond to specific digital treatment [22,23]. Personalized digital medicine will potentially boost the engagement, effect size, and effect duration levers of the clinical value equation, enabling higher price-points for apps to be justifiable based upon their higher clinical value. Although the existing literature may be used to estimate the clinical value delivered when apps are deployed in an untargeted fashion, estimates derived from the general literature should be seen as a lower bound to the potential that apps may deliver.

Monetary estimates of the economic value of the clinical benefits delivered by digital health apps to patients can be generated using a QALY-based approach involving values reported in the literature. Valuations are context-dependent and may change over time as apps are better targeted to specific populations of patients. Nonetheless, it is possible to produce estimates of the economic value of the clinical benefits that patients derive from apps using a universal framework.

## Abbreviations

EQ-5D: EuroQOL 5 dimensions
QALY: quality-adjusted life-year
